# Preparation and Properties of Attapulgite/Brucite Fiber-Based Highly Absorbent Polymer Composite

**DOI:** 10.3390/ma17081913

**Published:** 2024-04-20

**Authors:** Caihong Deng, Xinming Zhai, Wenrong Li, Qian Li, Rui Xiong, Fuyang Lu

**Affiliations:** 1 Qinghai Guoluo Highway Engineering Construction Co., Ltd., Xining 810008, China; 2 School of Materials Science and Engineering, Chang’an University, Xi’an 710061, China; xiongr61@126.com

**Keywords:** highly absorbent polymer, inorganic composite, swelling properties, attapulgite, brucite fiber

## Abstract

The ATP-BF-P(HEC-AA-AMPS) composite highly absorbent polymer was copolymerized with acrylic acid (AA) and 2-acrylamido-2-methylpropane sulfonic acid (AMPS) using an aqueous solution method with attapulgite (ATP) and attapulgite (ATP) as a matrix. The prepared ATP-BF-P(HEC-AA-AMPS) was characterized in terms of microstructure and tested for its water absorption capacity, water retention properties, and pH dynamic sensing ability. The results showed that the synthesized ATP-BF-P(HEC-AA-AMPS) had a rough and porous surface and a high water absorption capacity and rate, almost reaching the maximum water absorption around 20 min, and demonstrated excellent water retention performance at low and medium temperatures. ATP-BF-P(HEC-AA-AMPS) has a sensitive dynamic sensing ability in different pH solutions, with a high swelling capacity between pH 6.0 and 10.0. When the pH value exceeded 10.0, the swelling rate decreased rapidly. Additionally, the thermal stability and mechanical strength of the highly absorbent polymers were significantly improved after blending with ATP and BF.

## 1. Introduction

The shortage of freshwater resources is a significant concern in China and worldwide. In recent years, China’s rapid development and urbanization have significantly increased the demand for freshwater. In addition, a large amount of agricultural irrigation, industrial water use, domestic water use, etc., makes freshwater resources even scarcer. And at the same time, it produces a series of problems, such as water pollution, which has a significant impact on the sustainable development of the economy and society [1,2]. Therefore, how to use water scientifically, so that the limited water resources are efficiently and rationally applied, has aroused widespread concern among researchers. Highly absorbent polymer is a novel functional polymer material that comprises numerous hydrophilic groups and a mildly cross-linked network structure, resulting in outstanding water absorption and retention capabilities [3,4,5]. Highly absorbent polymers can absorb and retain a significant amount of water, even in challenging conditions such as specific pressure, saline, and alkaline environments [6]. Their weight often exceeds hundreds or even thousands of times when compared to conventional water-absorbent materials [7,8,9]. Thus, they are also known as super-absorbent or high-water-holding agents, and find wide applications in agriculture, forestry, gardening, construction, sanitary products, food, medicine, and other fields [10,11,12,13].

Highly absorbent polymers mainly include natural and modified highly absorbent polymers and synthetic highly absorbent polymers. Natural and modified polymers include starch, cellulose, and other natural products. Synthetic highly absorbent polymers include polyvinylates, polyvinyl alcohols, polyoxyethylene, etc. [14]. The first highly absorbent polymer was reportedly prepared by Fanta et al. [15] in 1971 at the Northern Research Institute of the U.S. Department of Agriculture using starch-grafted acrylonitrile. Highly water-absorbent polymers still need improvement due to increased production costs, an unfriendly environment, low gel strength, poor salt resistance, aggregation, and gel clogging [16,17,18]. Therefore, developing environmentally friendly, high-performance, and low-priced highly absorbent polymers is crucial.

Methods to improve the salt resistance of highly absorbent polymers mainly include the introduction of nonionic copolymeric monomers, such as acrylamide; the introduction of carboxyethyl cellulose and other macromolecular chains to form a semi-interpenetrating network structure; and the incorporation of inorganic components [19]. Methods to enhance the degree of cross-linking by raising the cross-linker content or applying surface cross-linking treatments raise the cost and reduce the salt water absorption of highly absorbent polymers at atmospheric pressure. In contrast, grafting inorganic components onto polymer chains can cost-effectively improve the water absorption multiplicity and mechanical properties of highly absorbent resins in ionic solutions, allowing them to adapt more effectively to more complex use environments [5,8]. The ATP surface contains many hydroxyl groups and exchangeable cations and has good dispersibility and hydrophilicity, which can improve the water absorption rate of superabsorbent polymers in ionic solutions. In addition, ATP and BF, as inorganic components, have much higher strength than polymer materials, which greatly helps to improve the mechanical strength of superabsorbent polymers.

To further improve the swelling capacity and mechanical strength of highly absorbent polymers, BF and ATP were used as a matrix to copolymerize ATP-BF-P(HEC-AA-AMPS) composite highly absorbent polymers with acrylic acid (AA) and 2-acrylamido-2-methylpropane sulfonic acid (AMPS). The materials were analyzed by combining characterization techniques such as FTIR, SEM, EDS, and thermal analysis structure and properties, and the samples’ pH-sensing ability and mechanical properties were tested. The introduced inorganic materials are cost-effective and can be used efficiently at a lower cost. The reaction time is shorter, and the process is more straightforward than that of the reversed-phase suspension polymerization method. This approach is suitable for large-scale industrial production.

## 2. Materials and Methods

### 2.1. Materials and Equipment

The experimental materials and equipment for this experiment are listed in Table 1 and Table 2. All experimental water was deionized water.

### 2.2. Preparation of ATP-BF-P(HEC-AA-AMPS) Highly Absorbent Polymer

**Figure 1:**
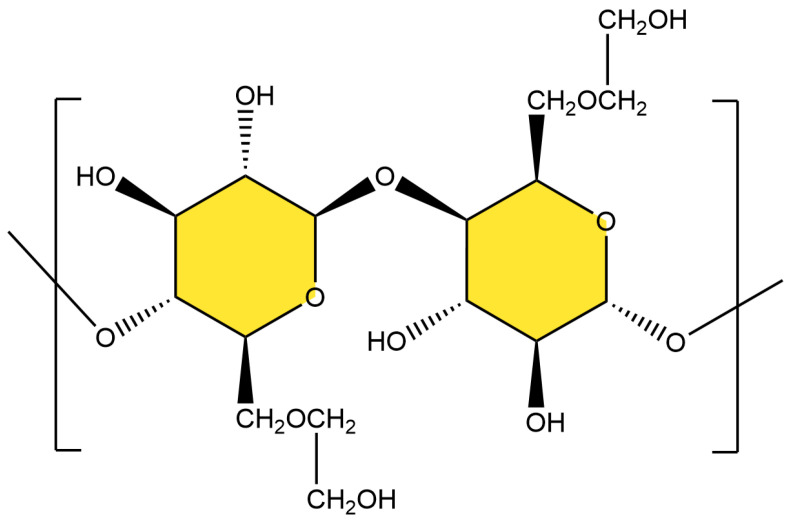
Schematic diagram of the molecular structure of HEC.

**Figure 2:**
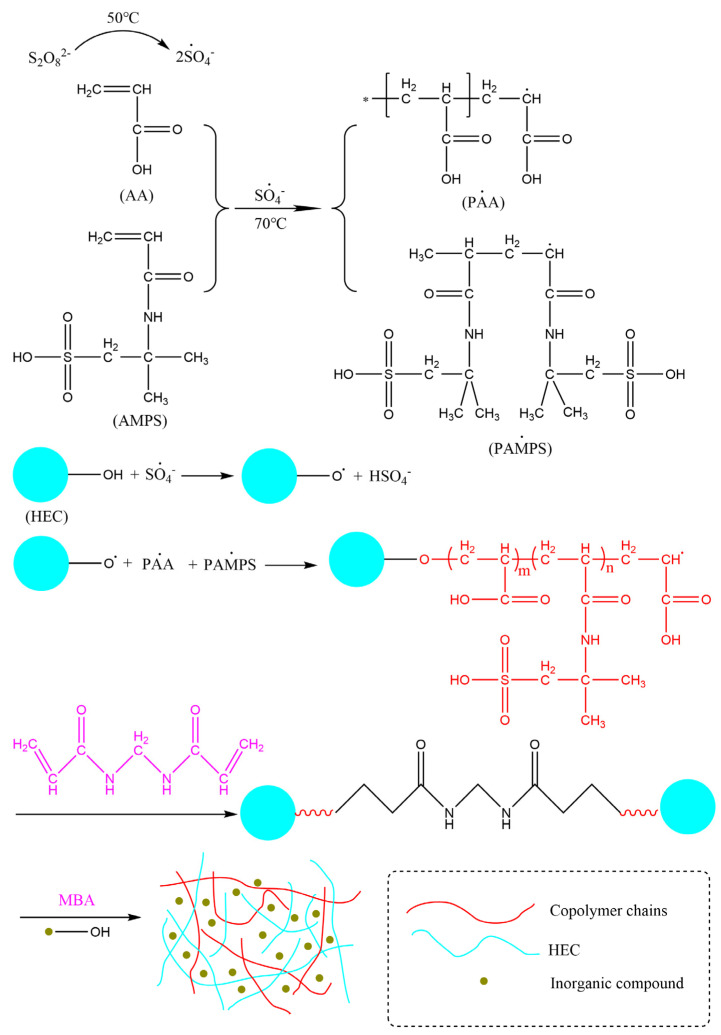
The reaction mechanism for synthesis of ATP-BF-P(HEC-AA-AMPS).

The appropriate amounts of 0.45 g HEC, 0.20 g ATP, 0.05 g SDS, 0.1 g BF, and 30 mL of deionized water were weighed and placed into a three-necked flask under nitrogen protection. The mixture was stirred 70 °C for 45 min to produce a dispersed emulsion, then cooled down to 40 °C, and 5 mL of 8 wt% APS solution was added dropwise. The mixture was stirred for 10 min, then 70% of neutralized AA, 1.42 g APMS, and 0.042 g MBA mixed solution were added. The temperature was raised to 70 °C and the reaction continued for three hours. After completion, the reaction mixture was soaked in ethanol solution for one hour to remove unreacted monomers. The product was transferred to a drying oven at 60 °C until a stable weight was achieved. The dried material was crushed with a pulverizer to produce ATP-BF-P(HEC-AA-AMPS) high-water-absorption polymer powder particles. Figure 1 and Figure 2 show the molecular structure of HEC and the reaction mechanism of the polymerization reaction.

### 2.3. Testing and Characterization

#### 2.3.1. SEM

To better understand the microscopic structure of ATP-BF-P(HEC-AA-AMPS) highly water-absorbent polymers, the surface morphology of the dried polymer powders was examined using a scanning electron microscope.

#### 2.3.2. EDS

The elemental content of the samples was measured using EDS analysis to determine whether ATP and BF were successfully introduced into the polymer.

#### 2.3.3. FT-IR

The solid sample was combined with KBr at a ratio of 1:100 by mass, then crushed into a fine powder and compressed into transparent sheets. The inferable spectra of the resulting samples were analyzed using a Fourier transform inferable spectrometer across the range of 4000–400 cm^−1^.

#### 2.3.4. Thermodynamic Analysis

The polymer was subjected to thermodynamic analysis utilizing a synchronized thermal analyzer test, conducted in a temperature range of 30 to 600 degrees Celsius with a temperature ramp of 10 degrees Celsius per minute, and carried out in a nitrogen-protected atmosphere.

#### 2.3.5. Measurement of Swelling Behavior

During the experiment, a custom filter bag was used to contain 0.1 g of the polymer sample, which was then submerged in a beaker filled with 500 mL of deionized water, tap water, a 0.9% NaCl solution, and a saturated Ca(OH)_2_ solution. The specimens were left to equilibrate at temperatures of 20 °C, 30 °C, 40 °C, and 50 °C. Following this, the polymer weight was recorded at intervals of 1, 2, 5, 7, 10, 15, 30, 60, 90, and 120 min. The water absorption ratio was then determined for each temperature by applying a specific equation.

Q = (M_2_ − M_1_)/M_1_ (1)

where M_1_ and M_2_ represent the mass of the dry and absorbent polymer, measured in grams, respectively; and Q stands for the water absorption multiplicity, measured in g/g [20].

#### 2.3.6. Determination of Water Retention at Different Temperatures

In the experiment, 0.1 g of the polymer sample was weighed, placed into deionized water to reach dissolution equilibrium, considered, and placed into an environmental chamber at 20 °C, 30 °C, 40 °C, and 50 °C. Weighing was performed at intervals, and the water retention multiplicity, R, was calculated at different temperatures using the equation:

R = (M_3_ − M_1_)/(M_2_ − M_1_) (2)

where M_1_ represents the mass of the dried polymer, measured in grams; M_2_ represents the mass of the polymer after reaching dissolution equilibrium, measured in grams; and M_3_ represents the mass of the polymer after water loss, measured in grams [21].

#### 2.3.7. pH Dynamic Perception Test

In this step, 0.1 g of polymer sample was weighed and immersed in 0.9% NaCl solution. The pH value, using hydrochloric acid and NaOH solution, was adjusted. Then, the swelling multiplication rates were measured at different pH values.

#### 2.3.8. Mechanical Test

The unformed polymer was placed into a 2 mL centrifuge tube and taken out after the polymerization process was complete. A cylindrical sample with a 10.4 mm diameter was created, and the compression characteristics of the water-absorbent polymer were analyzed using a mass spectrometer with a compression speed of 2 mm/s [22,23].

## 3. Results and Discussion

### 3.1. SEM

**Figure 3:**
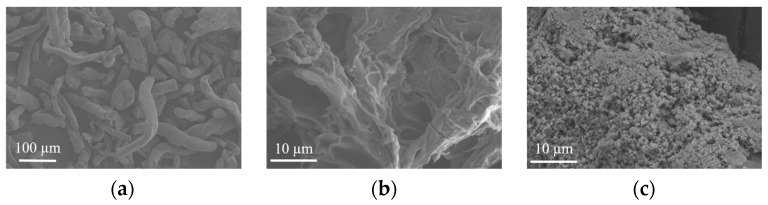
Scanning electron microscopy of (**a**) HEC, (**b**) P(HEC-AA-AMPS), and (**c**) ATP-BF-P(HEC-AA-AMPS).

SEM images of HEC, P(HEC-AA-AMPS), and ATP-BF-P(HEC-AA-AMPS) are presented in Figure 3, respectively. Hydroxyethyl cellulose (HEC) exhibited an extensive and substantial distribution with a relatively smooth surface, while the surface of P(HEC-AA-AMPS) featured a porous structure with smooth pore walls and an uneven size distribution. Following the incorporation of ATP and BF, the surface of the highly absorbent polymer underwent substantial roughening, featuring numerous tiny pores and significant porosity, signifying the successful involvement of ATP and BF in the polymerization reaction. The porous structure facilitated the infiltration of water molecules into the interior of the material’s three-dimensional network, thereby enhancing its water absorption properties.

### 3.2. EDS

**Figure 4:**
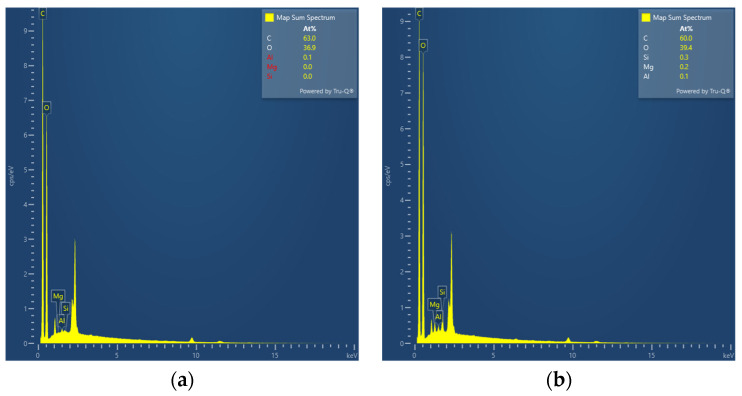
EDS spectra of (**a**) P(HEC-AA-AMPS) and (**b**) ATP-BF-P(HEC-AA-AMPS).

P(HEC-AA-AMPS) and ATP-BF-P(HEC-AA-AMPS) were subjected to EDS tests at the same scanning magnification mainly to measure the changes in the content of the elements C, O, Al, Mg, and Si. Table 3 shows the results of the elemental tests, and Figure 4 shows the EDS energy spectra. It was found that the content of Mg and Si elements in the modified highly absorbent polymer increased significantly, indicating that ATP and BF were successfully doped into the highly absorbent polymer.

### 3.3. FT-IR

**Figure 5:**
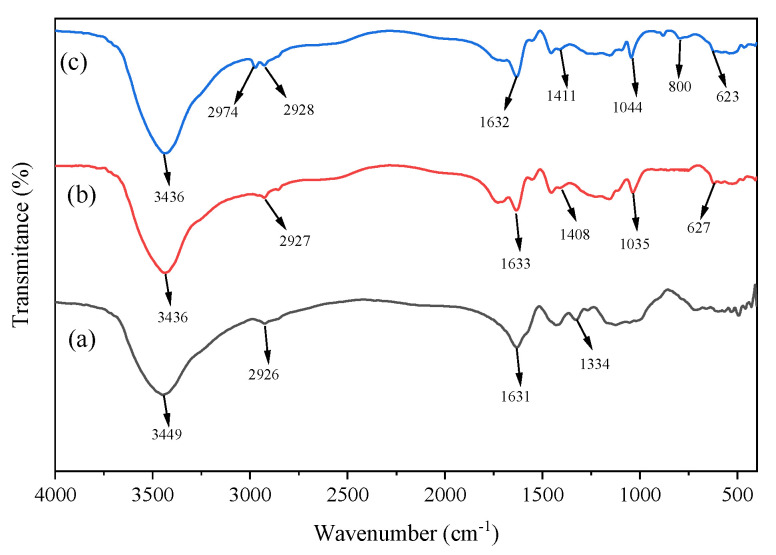
FTIR spectra of (**a**) HEC, (**b**) P (HEC-AA-AMPS), and (**c**) ATP-BF-P(HEC-AA-AMPS).

In Figure 5, the infrared spectra of hydroxyethyl cellulose (HEC), P(HEC-AA-AMPS), and ATP-BF-P(HEC-AA-AMPS) are shown. The infrared spectrum of HEC is displayed in curves a, showing the flat vibrational peak of C-OH at 1334 cm^−1^. The distinct peak at 2926 cm^−1^ is assigned to the stretching vibration of aliphatic C-H in HEC [24], which is also apparent in graphs b and c. The anticipated peak at 3449 cm^−1^, corresponding to the telescopic vibration of O-H on HEC, exhibits lower intensity in comparison to curves b and c. This phenomenon arose because the characteristic peaks in curves b and c resulted from the combined effects of O-H and N-H stretching vibrations [25,26]. Within curves b and c, distinctive features include the telescopic vibration peaks of O=S at 627 cm^−1^ and 623 cm^−1^, the asymmetric telescopic vibration peaks of C-O-C at 1035 cm^−1^ and 1044 cm^−1^, and the asymmetric telescopic vibration peaks of COO- at 1408 cm^−1^ and 1411 cm^−1^. These characteristic peaks signify the successful polymerization of P(AA) and P(AMPS) into the HEC backbone [27]. Additionally, the peaks observed at 800 cm^−1^ in curve c correspond to the bending vibrational peaks of Si-O-Si, while the 2974 cm^−1^ peaks for C-H stretching in BF confirm the effective incorporation of inorganic materials into the HEC polymer [28].

### 3.4. Thermal Stability Analysis

**Figure 6:**
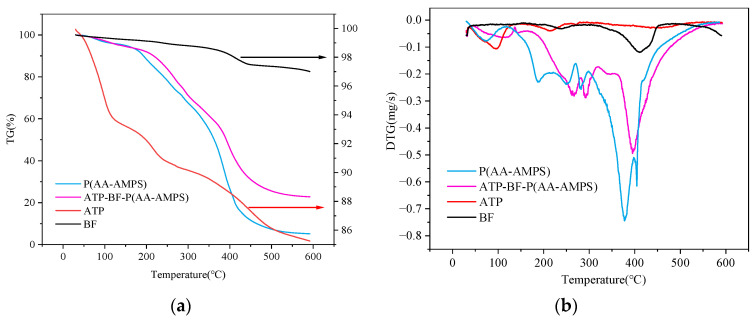
(**a**) TG curves and (**b**) DTG curves of ATP, BF, P(HEC-AA-AMPS), and ATP-BF-P(HEC-AA-AMPS).

The TG and DTG curves of ATP, BF, P(HEC-AA-AMPS), and ATP-BF-P(HEC-AA-AMPS) are shown in Figure 6. From the figure, the thermal stability of BF and ATP was much higher than that of organic materials, with only 17.47% and 14.7549% mass loss at 600 °C. The thermal decomposition process of P(HEC-AA-AMPS) and ATP-BF-P(HEC-AA-AMPS) was divided into four stages, corresponding to temperatures of 30 to 170 °C, 170 to 340 °C, 340 to 417 °C, 417 to 600 °C, 30 to 200 °C, 200 to 374 °C, 374 to 467 °C, and 467 to 600 °C. the temperatures corresponding to the maximum decomposition rate were 377 °C and 395 °C, respectively. The decomposition process of composite water-absorbing materials unfolded across distinct stages. The first stage primarily involved the decomposition of water and unreacted raw materials within these composites. Significant decomposition took place primarily in the second and third stages. The second stage involved the decomposition of small molecules, like oligomers, present in the material, while the third stage was characterized by the decomposition of branched chains within the polymer. Subsequently, in the fourth stage, mass reduction took place as a result of the breakdown of the polymer’s main chains and the disintegration of its three-dimensional network structure.

Comparing the TG plots of P(HEC-AA-AMPS) and ATP-BF-P(HEC-AA-AMPS), it can be found that the thermal stability of ATP-BF-P(HEC-AA-AMPS) was significantly better than that of the second one in the remaining stages, except for the first stage, which was similar to that of P(HEC-AA-AMPS), and the remaining sample masses of the two after 600 °C were, respectively, 5.18% and 22.76%. These findings demonstrate that ATP and BF play a role in the polymerization process and enhance the thermal stability of the materials.

### 3.5. Measurement of Swelling Behavior

The swelling multiplier and swelling rate are critical performance indicators of high-absorbency polymers, serving as the technical prerequisites for determining their suitability for widespread use.

**Figure 7:**
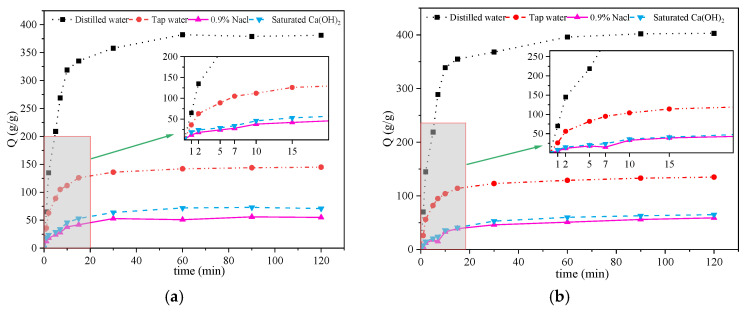

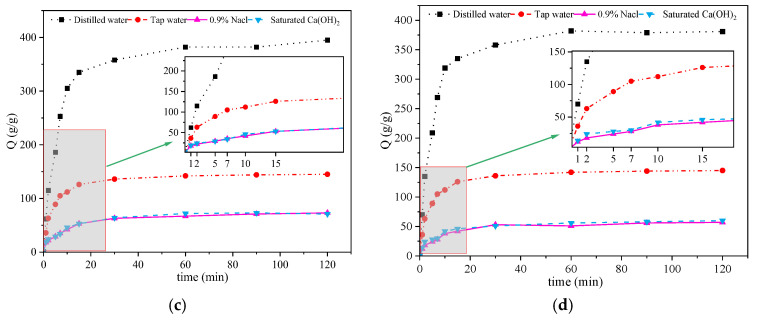
Swelling performance test at different temperatures: (**a**) 20 °C, (**b**) 30 °C, (**c**) 40 °C, and (**d**) 50 °C.

Figure 7 displays the outcomes of the swelling experiment for ATP-BF-P(HEC-AA-AMPS) at temperatures of 20 °C, 30 °C, 40 °C, and 50 °C. The swelling rate of ATP-BF-P(HEC-AA-AMPS) in deionized water demonstrated a trend of initially increasing and then decreasing as the temperature rose, peaking at a maximum water-absorbing capacity of 403 g/g at 30 °C. Beyond this temperature, the water absorption capacity started to decline. Notably, in both 0.9% NaCl solution and saturated Ca(OH)_2_ solution, the water absorption capacity of ATP-BF-P (HEC-AA-AMPS) increased from 59 g/g and 65 g/g, respectively, to 73 g/g and 71 g/g as the temperature increased from 30 °C to 40 °C. This could be explained by the observation that the water absorption rate of the ATP-BF-P(HEC-AA-AMPS) three-dimensional structure seemed to be somewhat disrupted, allowing for the entry of ions and resulting in an enhanced water absorption capacity in the presence of an ionic solution.

However, with increasing temperature, the degree of destruction of the three-dimensional network structure intensified, leading to a decrease in the water-absorbing capacity of ATP-BF-P(HEC-AA-AMPS) in ionic solution. Specifically, at 50 °C, the water-absorbing capacities in deionized water, 0.9% NaCl solution, and saturated Ca(OH)_2_ solution were 381 g/g, 145 g/g, 57 g/g, and 60 g/g. These values represent decreases of 22 g/g, 10 g/g, 3 g/g, and 5 g/g compared to those observed at 30 °C, with minimal performance variation.

In addition, in the range of 20 to 50 °C, the prepared samples all reached the expansion equilibrium state within 15 to 20 min, and the prepared samples met the performance requirements of rapid dissolution and swelling under the medium-to-low temperature environment.

**Figure 8:**
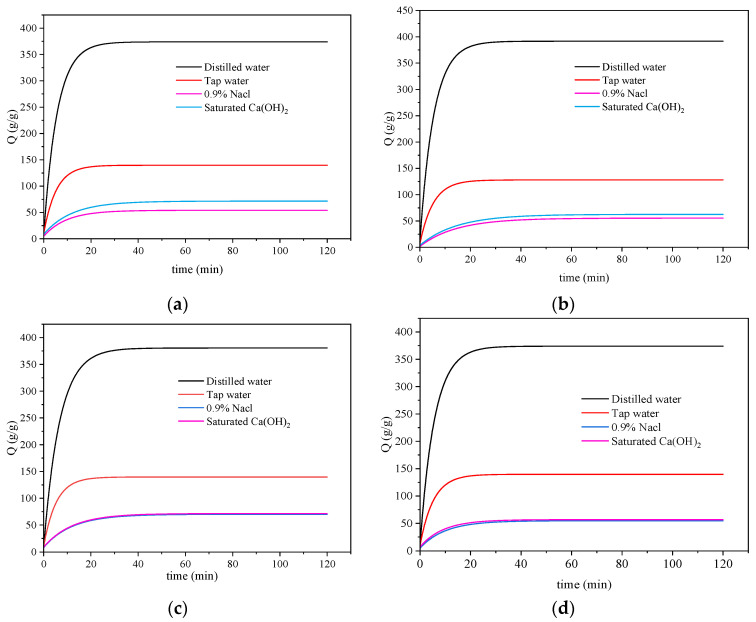
Swelling performance fitting curve: (**a**) 20 °C, (**b**) 30 °C, (**c**) 40 °C, and (**d**) 50 °C.

The solubilization data of the prepared samples at various temperatures underwent nonlinear curve fitting and were plotted as depicted in Figure 8. The corresponding fitted curve equations and R^2^ values are presented in Table 4. It is evident that the fitted curve closely approximates the actual data, with the lowest R^2^ value of 0.95434 observed in the saturated Ca(OH)_2_ solution at 50 °C.

### 3.6. Water Retention Properties

In the case of highly absorbent resins, prioritizing strong water retention properties over high swelling multiplicity is crucial. This is exemplified in soil moisturizers and concrete internal curing agents, where a slow water release is essential for maintaining prolonged humidity within soil and concrete structures.

**Figure 9:**
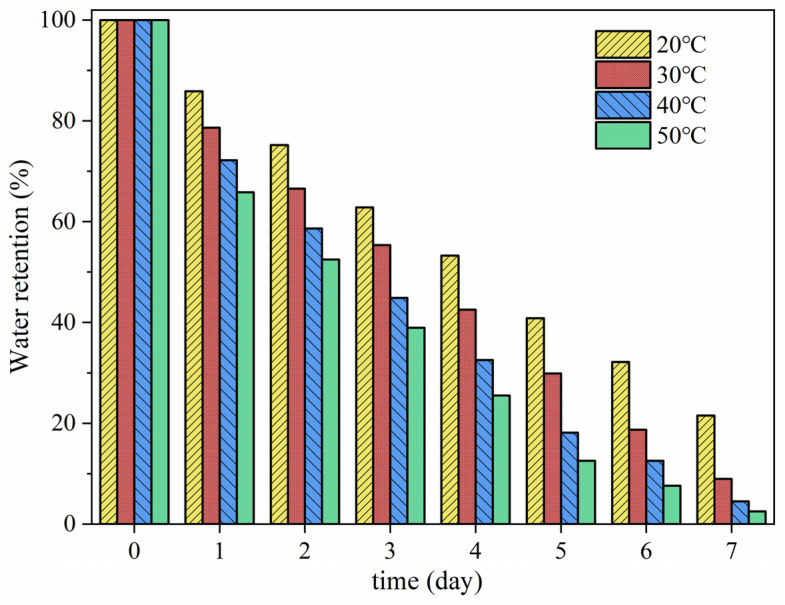
Water retention performance test.

In order to assess the water retention capability of the material ATP-BF-P(HEC-AA-AMPS), it was dissolved until reaching equilibrium at room temperature. Afterward, the water was extracted and moved to a stable-temperature environment in chambers set at 20 °C, 30 °C, 40 °C, and 50 °C, with the weight variations recorded for a period ranging from 1 to 7 days. The water-holding capacity was determined based on Equation (2), and the findings are shown in Figure 9. The water retention performance significantly decreased with increasing temperature, possibly due to the increased water evaporation at higher temperatures. Additionally, the decrease in water retention may have been caused by the deterioration of the resin structure due to prolonged exposure to high temperatures, resulting in a lower ability to absorb water. The water retention rates of the samples at 20°C, 30°C, 40°C, and 50°C on the initial day were 85.91%, 78.65%, 72.18%, and 65.86%. The difference in water retention between 20 °C and 50 °C was 20.05%. Over the course of the testing period, the differences between these temperatures on the 2nd to 7th days increased and then decreased, with values of 22.68%, 23.88%, 27.74%, 28.3%, 24.56%, and 19.03%, respectively. After 7 days of experimentation, the water retention rates under environments of 20 °C, 30 °C, 40 °C, and 50 °C were 23.61%, 9.82%, 4.32%, and 2.36%, respectively, demonstrating the material’s favorable water retention performance under medium-to-low temperature conditions.

Additionally, since the test was conducted in a controlled environment, it’s worth noting that in practical applications, high-water-absorption resin is frequently encapsulated within other materials. For instance, it may be used as a soil moisturizer mixed into dry soil, or as a concrete internal curing agent incorporated inside the concrete structure. This implies that, in real-world scenarios, the water retention effect is likely to be even more pronounced.

### 3.7. pH Dynamic Sensing

**Figure 10:**
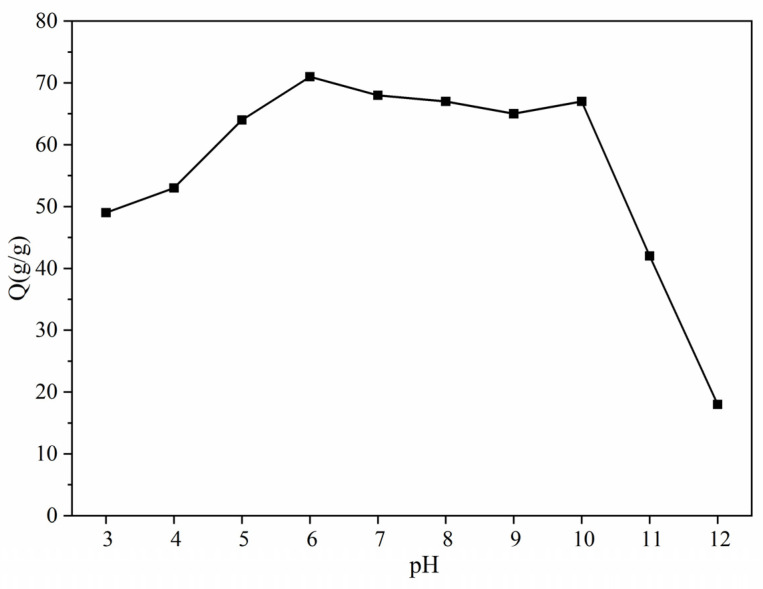
Dissolution multiplicity of samples as a function of pH value.

Figure 10 illustrates the variation curves of the dissolution test for the highly absorbent polymer in a 0.9% NaCl solution at various pH levels. In order to minimize or eliminate variations in ionic strength resulting from significant changes in factors like solution volume, the pH of the solution was adjusted using 11.5 mol/L HCl or NaOH.

Figure 7 illustrates that ATP-BF-P(HEC-AA-AMPS) achieved a maximum swelling capacity of 71 g/g at a pH of 6.0. The swelling diversity of the samples exhibited a more rapid increase as the pH rose from 3.0 to 6.0. Between pH 6.0 and 10, the swelling diversity exhibited no significant change, but rapidly decreased as the pH increased. The swelling behavior of highly absorbent resins can vary in different pH environments due to factors such as hydrogen bonding, electrostatic repulsion, and coordination along the resin chain. Conversely, electrostatic repulsion and high osmotic pressure contribute to increases in the swelling diversity of the highly absorbent polymers [29,30,31]. At pH levels below 3.0, the H^+^ in the swelling medium will exchange with the Na^+^ in the ATP-BF-P(HEC-AA-AMPS) polymer. Simultaneously, the presence of numerous carboxyl groups on the highly absorbent polymer leads to hydrogen bonding between these groups, resulting in a lower swelling multiplication rate. As the pH rises, more acid ions join the chain, leading to increased electrostatic repulsion within the highly absorbent resin network. This, combined with heightened osmotic pressure inside and outside the structure, results in the resin network expanding fully and the equilibrium water absorption rising. While the coordination ability of the highly absorbent resin also increases and the equilibrium water absorption decreases, the primary factors driving the enhanced absorption remain the electrostatic repulsion within the macromolecule chain and the heightened osmotic pressure inside and outside the network. Consequently, the equilibrium water absorption continues to increase [32,33].

When the pH exceeds 6.0, the electrostatic repulsion on the polymer chain diminishes, impeding network expansion. Simultaneously, the coordination ability between polymer chains strengthens [34]. Consequently, the swelling capacity begins to decrease. Moreover, a further increase in the amount of Na^+^ reduces the oxygen–sodium ratio on the polymer chain. This leads to a decrease in the coordination between sodium and oxygen, causing a shift in the coordination mode from intramolecular to intermolecular. Consequently, the network of the highly water-absorbent polymer expands further. The comprehensive results indicate that the equilibrium water absorption of the highly water-absorbent polymer remains constant as the pH increases from 7.0 to 10.0. Beyond a pH of 10, the water absorption initiates a decline, potentially attributable to low osmotic pressure and diminished electrostatic repulsion.

### 3.8. Mechanical Performance Test

Highly absorbent polymers undergo extrusion and friction during usage, leading to destructive damage that impacts their internal structure. Hence, they must possess a certain level of mechanical strength. To ensure experimental rigor, the prepared samples underwent washing with ethanol and deionized water before being directly subjected to a stress–strain test.

**Figure 11:**
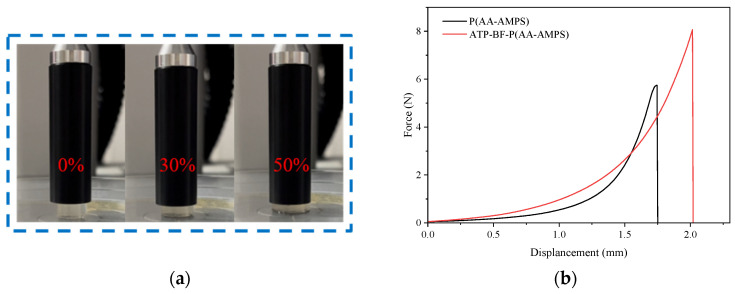
(**a**) Photograph of the test site; (**b**) stress–strain curve.

Figure 11a displays field test images of ATP-BF-P(HEC-AA-AMPS) at 0%, 30%, and 50% compressive deformation levels, illustrating a gradual thinning of the samples which was observed visually. Subsequently, Figure 11b presents stress–strain curves of P(HEC-AA-AMPS) and ATP-BF-P(HEC-AA-AMPS) at a 50% deformation level. The graphs indicate that the maximum positive stress of the water-absorbing polymers increased from 5.74 N to 8.07 N with the addition of ATP and BF, resulting in a notable 40.59% improvement in mechanical properties. Additionally, at the same level of positive stress, the deformation of the modified polymer was smaller. For instance, the deformation before and after modification was 1.631 mm and 1.701 mm at 4 N, corresponding to compressions of 46.71% and 42.19%, respectively. This demonstrates that the incorporation of ATP and BF enhances the strength and stability of the polymer structure, rendering it more suitable for application in challenging environments.

## 4. Conclusions

(1) The successful polymerization of ATP-BF-P(HEC-AA-AMPS), a high-absorbency polymer, using an aqueous solution was demonstrated through SEM and FTIR. The polymer exhibited increased roughness and a higher number of micropores after the introduction of ATP and BF, significantly enhancing its liquid-absorbing capacity.
(2) ATP-BF-P(HEC-AA-AMPS) exhibited superior thermodynamic stability in the temperature range of 30–600 °C compared to P(HEC-AA-AMPS), with a 17.58% reduction in mass loss at 600 °C, signifying a noteworthy improvement in thermal stability.
(3) ATP-BF-P(HEC-AA-AMPS) exhibited better adaptability to the pH range, with minimal changes in dissolution multiplicity and maximum water absorption multiplicity between pH 6.0~10.0, indicating a broad range of applicability. Conversely, beyond a pH value of 10.0, the ability to absorb liquids decreased rapidly.
(4) The mechanical properties of ATP-BF-P(HEC-AA-AMPS) improved by 40.59% at a 50% deformation level with the addition of ATP and BF. The overall strength of the polymer was significantly enhanced, rendering it more suitable for use in complex scenarios.

## Figures and Tables

**Table 1:** Experimental reagents.

Reagents	Abbreviation	Reagent Purity	Manufacturer
Acrylic acid	AA	Analytical purity	Tianjin Damao Chemical Reagent Factory (Tianjin, China)
2-Arylamido-2-methylpropane sulfonic acid	AMPS	Analytical purity	Shanghai McLean Biochemical Technology Co., Ltd. (Shanghai, China)
Brucite fiber	BF	/	Provinsi Shaanxi (Yulin, China)
Attapulgite	ATP	/	Provinsi Shaanxi
Methylene-bis-acrylamide	MBA	Analytical purity	Shanghai McLean Biochemical Technology Co., Ltd.
Carboxyethyl cellulose	HEC	Analytical purity	Shanghai McLean Biochemical Technology Co., Ltd.
Calcium hydroxide	Ca(OH)_2_	Analytical purity	Tianjin Damao Chemical Reagent Factory (Tianjin, China)
Sodium chloride	NaCl	Analytical purity	Tianjin Damao Chemical Reagent Factory
Ethanol absolute	C_2_H_6_O	Analytical purity	Tianjin Damao Chemical Reagent Factory
Ammonium persulphate	APS	Analytical purity	Shanghai Aladdin Biochemical Science and Technology Co. (Shanghai, China)
Potassium bromide	KBr	Analytical purity	Shanghai McLean Biochemical Technology Co., Ltd.

**Table 2:** Experimental equipment.

Equipment	Model	Manufacturer
Fourier Transform Infrared Spectrometer	TENSOR II	Bruker (Beijing) Technology Co. (Beijing, China)
Synchronous Thermal Analyzer	STD650	Waters China Ltd. (Kowloon, Hong Kong)
Scanning Electron Microscope	S-4800	Hitachi Production Co., Ltd. (Tokyo, Japan)
Mass Structural Analyzer	TA.TOUCH	Shanghai Baosheng Industrial Development Co., Ltd. (Shanghai, China)
Magnetic Water Bath Stirrer	DF-101S	Henan Iuka Instrument Co. (Zhengzhou, China)
Henan Iuka Instrument	WGL-45B	Tianjin Tester Instrument Co. (Tianjin, China)

**Table 3:** EDS elemental content test results.

Element	C (At%)	O (At%)	Al (At%)	Mg (At%)	Si (At%)
P(HEC-AA-AMPS)	63.0	36.9	0.1	-	-
ATP-BF-P(HEC-AA-AMPS)	60.0	39.4	0.1	0.2	0.3

**Table 4:** Fitting curve equations.

Temperature	Solution	Equation	R^2^
20 °C	Deionized water	Q = 373.93816 − 366.51695 ∗ 0.83853^t^	0.99228
	Tap water	Q = 139.67793 − 129.44161 ∗ 0.827623^t^	0.97808
	0.9% NaCl solution	Q = 54.12796 − 49.18642 ∗ 0.90225^t^	0.97906
	Ca(OH)_2_ solution	Q = 71.51842 − 62.77467 ∗ 0.91987^t^	0.97126
30 °C	Deionized water	Q = 391.48654 − 384.12705 ∗ 0.83314^t^	0.98907
	Tap water	Q = 128.06336 − 122.2181 ∗ 0.82654^t^	0.97903
	0.9% NaCl solution	Q = 55.48576 − 53.88255 ∗ 0.93325^t^	0.96452
	Ca(OH)_2_ solution	Q = 62.66125 − 59.10752 ∗ 0.93333^t^	0.98705
40 °C	Deionized water	Q = 380.6345 − 372.91741 ∗ 0.86263^t^	0.99273
	Tap water	Q = 139.67793 − 129.44161 ∗ 0.82762^t^	0.97808
	0.9% NaCl solution	Q = 70.0173 − 61.89942 ∗ 0.91964^t^	0.97509
	Ca(OH)_2_ solution	Q = 71.51842 − 62.77467 ∗ 0.91987^t^	0.97126
50 °C	Deionized water	Q = 373.96533 − 363.99687 ∗ 0.8393^t^	0.99229
	Tap water	Q = 139.67793 − 129.54161 ∗ 0.82762^t^	0.97808
	0.9% NaCl solution	Q = 54.71655 − 49.6725 ∗ 0.90467^t^	0.97741
	Ca(OH)_2_ solution	Q = 56.77377 − 49.76065 ∗ 0.89635^t^	0.95434

## Data Availability

The data used to support the findings of this study are included within the article.
